# Pharmacokinetic and Pharmacodynamic Rationale for Extending VEGF Inhibition Increasing Intravitreal Aflibercept Dose

**DOI:** 10.3390/pharmaceutics15051416

**Published:** 2023-05-06

**Authors:** Daniele Veritti, Valentina Sarao, Francesco Di Bin, Paolo Lanzetta

**Affiliations:** 1 Department of Medicine–Ophthalmology, University of Udine, 33100 Udine, Italy; 2 Istituto Europeo di Microchirurgia Oculare (IEMO), 33100 Udine, Italy

**Keywords:** age-related macular degeneration, aflibercept, burden, intravitreal injections, mathematical model, neovascular

## Abstract

Background: The effects of various dosages and treatment regimens on intravitreal aflibercept concentrations and the proportion of free vascular endothelial growth factor (VEGF) to total VEGF were evaluated using a drug and disease assessment model. The 8 mg dosage received specific attention. Methods: A time-dependent mathematical model was developed and implemented using Wolfram Mathematica software v12.0. This model was used to obtain drug concentrations after multiple doses of different aflibercept dosages (0.5 mg, 2 mg, and 8 mg) and to estimate the time-dependent intravitreal free VEGF percentage levels. A series of fixed treatment regimens were modeled and evaluated as potential clinical applications. Results: The simulation results indicate that 8 mg aflibercept administered at a range of treatment intervals (between 12 and 15 weeks) would allow for the proportion of free VEGF to remain below threshold levels. Our analysis indicates that these protocols maintain the ratio of free VEGF below 0.001%. Conclusions: Fixed q12–q15 (every 12–15 weeks) 8 mg aflibercept regimens can produce adequate intravitreal VEGF inhibition.

## 1. Introduction

Age-related macular degeneration (AMD) is a degenerative eye disorder that affects the macula, the central part of the retina responsible for central vision. It is the primary cause of irreversible vision impairment and blindness in the developed world. As the global population ages, the prevalence of AMD is forecasted to increase from 170 to 288 million people by 2040 [1]. Neovascular AMD accounts for the majority of cases of blindness and it is primarily caused by abnormal new blood vessel growth in the eye, also known as choroidal neovascularization [2]. These new blood vessels are fragile and may leak fluid and blood into the macula, resulting in distorted and blurred vision. In the most advanced cases, vision loss can be so severe that a patient may become legally blind. The introduction of vascular endothelial growth factor (VEGF) inhibitors has revolutionized the prognosis of neovascular AMD patients, becoming the cornerstone of therapy for this condition [3,4]. However, owing to the limited half-life of these drugs, frequent intravitreal injections are required, resulting in a significant burden on patients, caregivers, and healthcare systems. In an effort to reduce the burden of frequent injections, strategies such as developing new drugs targeting multiple pathological pathways, agents with longer half-lives, and controlled-release devices have been proposed [5,6,7,8]. One example of a dual-target drug is faricimab (Vabysmo^®^, Genentech, San Francisco, CA, USA and Roche, Basel, Switzerland), which is a bispecific antibody designed to target both VEGF-A and angiopoietin-2 (Ang-2). The dual mechanism of action of faricimab targets two key pathways implicated in the pathogenesis of neovascular AMD and diabetic macular edema, providing a longer duration of action. Another novel drug that has been developed to reduce the injection frequency required for treatment is brolucizumab (Beovu^®^, Novartis Pharma AG, Basel, Switzerland), a single-chain antibody fragment that selectively inhibits VEGF-A. Its small size enables higher molar dosing, which, combined with its high binding affinity to VEGF-A, contributes to its increased durability [5,6]. A further strategy is to increase the dosage of a drug that is already in use. The HARBOR trial was the first study to evaluate the safety and efficacy of higher doses of the established anti-VEGF agent ranibizumab (Lucentis^®^, Novartis Pharma AG, Basel, Switzerland and Genentech, San Francisco, CA, USA). The study compared intravitreal ranibizumab at 0.5 mg and 2.0 mg administered monthly and on an as-needed basis. The average treatment intervals were 9.9 weeks in the 0.5 mg PRN group and 12.5 weeks in the 2 mg PRN group [9]. Aflibercept (Eylea^®^, Regeneron Pharmaceuticals, Tarrytown, NY, USA and Bayer HealthCare, Berlin, Germany) is a recombinant fusion protein approved for the treatment of ocular conditions, including neovascular AMD. As a VEGF inhibitor, it inhibits the growth of abnormal new vessels and reduces vascular permeability. The approved dose of aflibercept for the treatment of neovascular AMD is 2 mg (0.05 mL) administered via an intravitreal injection. To assess the efficacy and safety of a higher dose of aflibercept (8 mg) in neovascular AMD, the PULSAR trial was conducted [10]. This was based on the rationale that a higher dose of medication may have longer durability. The PULSAR trial was a phase 3 active-controlled trial that compared the efficacy and safety of a high dose of aflibercept (8 mg) injected with longer intervals (12 and 16 weeks) with the standard 2 mg dose administered every 8 weeks. Analysis of the data showed that 83% of the patients treated with a high dose of aflibercept were able to maintain at least a 12-week interval between treatments, with no significant difference in the best-corrected visual acuity (BCVA) outcomes when compared with the control group. The safety profile of aflibercept 8 mg was reported to be comparable to that of the standard 2 mg dose group, with no differences in intraocular pressure and no cases of occlusive retinal vasculitis or endophthalmitis [10]. These findings suggest that a higher dose of aflibercept could be administered as a safe and effective alternative to the standard dose in neovascular AMD, with less frequent injections than the standard 2 mg dose. In the present paper, we present a pharmacokinetic (PK) and pharmacodynamic (PD) rationale for extending the dosing interval of intravitreal aflibercept by increasing the dose from 2 mg to 8 mg. Using a previously published and validated mathematical model [11], we sought to simulate the effect of augmenting the dose on aflibercept drug dynamics after intravitreal administration and to determine whether this could lead to an extended dosing interval that would reduce the burden of frequent injections while maintaining the therapeutic effect. The model validation was performed in a previous study [11] that evaluated the pharmacokinetic and pharmacodynamic properties of anti-VEGF drugs, including 0.5 mg ranibizumab, 1.25 mg bevacizumab, 2 mg aflibercept, and 6 mg brolucizumab, administered under various treatment regimens and compared the proportions of unbound VEGF predicted by the model with clinical efficacy data.

## 2. Materials and Methods

A systematic literature review was conducted to identify and gather PK and PD information for aflibercept in human eyes. The results of the most pertinent trials regarding the treatment of neovascular AMD were also compiled. An extensive search of major electronic databases was conducted to identify clinical trials relevant to the scope of this evaluation. The databases searched included EMBASE, PubMed, and Cochrane, with the last research conducted in December 2022 by two reviewers (VS and DV). The search methodology was focused on the integration of Medical Subject Headings (MeSH) and the keywords “age-related macular degeneration”, “choroidal neovascularization”, “anti-VEGF”, “AMD”, “CNV”, and “aflibercept”. The review was restricted to peer-reviewed papers, English language, and those published until 2022. The two reviewers independently assessed the studies for eligibility, and the resolution of discrepancies was achieved through deliberation with a third reviewer (PL). In order to ensure a thorough and comprehensive search of the literature, the reference lists of the retrieved articles were manually searched for additional pertinent studies. The titles of the identified studies were then reviewed and only papers that could potentially provide relevant information were obtained. Additionally, all available abstracts were reviewed and published systematic reviews, editorials, and reviews were also consulted. This comprehensive search strategy was intended to identify all relevant studies and minimize the risk of bias and omission of important studies.

A mathematical model was developed and evaluated to determine the time-dependent intravitreal concentrations of the drug and the ratio of VEGF not bound to aflibercept after intravitreal administration. We simulated the effects of various dosages of aflibercept (0.5 mg, 2 mg, and 8 mg). Mathematica version 12.0 (Wolfram Research, Inc., Champaign, IL, USA) was utilized to generate graphs of the intravitreal aflibercept concentration and free VEGF proportion. A unidirectional, single-chamber model was utilized to characterize the pharmacokinetic removal of the compound from the vitreous humor, with the molar concentrations of the substance determined in accordance with the administered dosage. The parameters for the model were derived from a targeted literature review and included the intravitreal half-life of aflibercept (9.1 days), the dissociation constant between aflibercept and VEGF-A165 (0.49 pM), and the molecular mass (115 kDa) [12,13,14].

The differential equation that describes the drug concentration in the vitreous at a certain time after a certain dosage and number of doses used for the present model is as follows:

(1) $$x\left(t\right)=-\frac{\log(2)}{\tau }x\left(t\right)+\frac{d}{4}\sum_{k=0}^{n-1}\delta \left(t-k\tau \right)$$

where:

− x(t) is the drug concentration at time t;
− t is time;
− δ is the Dirac delta function (impulsive jolt);
− d is the dose;
− n is the total number of injections;
− τ is the interval between injections.

The equation employed to delineate the fraction of unbound VEGF was:

(2) $$Kd=\frac{{\left[A\right]}^{x}{\left[B\right]}^{y}}{\left[AxBy\right]}$$

where:

− Kd represents the dissociation constant;
− [A]^x^, [B]^y^, and [AxBy] are the molar concentrations of drug subunits, VEGF subunits, and the complex, respectively.

The initial vitreous molar drug concentrations were calculated using a vitreous volume of 4 mL. Flip-flop pharmacokinetic relationships for each drug were assumed, with the slower process dominating the kinetics. In order to determine a suitable threshold level for free VEGF, a previous study was considered [11]. The analysis used a drug and disease assessment model to evaluate the impacts of different treatment regimens on the intravitreal ranibizumab, bevacizumab, aflibercept, and brolucizumab concentrations and the proportion of free VEGF to total VEGF. Clinical outcomes from various published reports were combined and juxtaposed with free VEGF spikes that were inferred from the model. The results of this comparison suggested that a free VEGF threshold level of 0.001% could be appropriate. Further validation was provided by Muether et al., who analyzed the temporal correlations of VEGF suppression, recurrence of neovascular AMD, and visual acuity loss following intravitreal ranibizumab [15].

## 3. Results

### 3.1. Targeted Literature Review

Aflibercept, a 115 KDa fully human recombinant fusion protein, consists of extracellular domains 2 and 3 from the receptors VEGFR1 and VEGFR2, respectively, conjoined to the Fc region of human IgG1. This protein was engineered by integrating the amino acid sequences from the key binding domains of two human VEGF receptors into a human IgG-1 Fc structure. Aflibercept demonstrates a strong affinity for VEGF A, B, and PlGF (placental growth factor) [16,17]. Approval was granted by the United States Food and Drug Administration for the treatment of neovascular AMD in 2011 and by the European Medicines Agency in 2012 following the completion of the phase III VIEW trials, which evaluated the safety and efficacy of 2 mg aflibercept. In the VIEW study program, a total of 2457 participants were randomly divided into four groups: 0.5 mg aflibercept every 4 weeks (0.5q4), 2 mg aflibercept every 4 weeks (2q4), 2 mg aflibercept every 8 weeks (2q8) following three monthly loading injections, and 0.5 mg ranibizumab every 4 weeks (Rq4). The primary study period lasted for 52 weeks, followed by a follow-up period of 44 weeks. During the follow-up, all groups transitioned from a fixed regimen to a capped, as-needed regimen. The findings showed that 2q8 was non-inferior to monthly ranibizumab and maintained its efficacy at 96 weeks. Patients in the 2q8 group gained an average of +7.6 letters from baseline at week 96 (+8.4 letters at week 52), while the ranibizumab group gained an average of +7.9 letters from baseline at week 96 (+8.7 letters at week 52). Over the course of 2 years, patients in the 2q8 aflibercept group received an average of 11.2 injections and patients in the 0.5q4 ranibizumab group received 16.5 injections [18]. Aflibercept has also been investigated for its PK and PD properties. Pharmacokinetic investigations have indicated that, following the intravitreal administration of 2 mg aflibercept, its VEGF binding capacity at 83 days post-injection is analogous to the activity exhibited by ranibizumab at the 30-day mark. A prospective case series of five eyes with neovascular AMD assessed the half-life of aflibercept in aqueous humor after a single intravitreal injection, showing a median peak concentration of free aflibercept of 122 mg/L and a mean half-life of 9.1 days in the eye [12]. Aflibercept has a much stronger binding affinity to the VEGF receptor (Kd = 0.49 pmol/L) in comparison to ranibizumab (Kd = 46 pmol/L) and bevacizumab (Kd = 58 pmol/L), with a difference of nearly 100-fold [13]. Moreover, it is unique in its ability to inhibit VEGF-A, PlGF-1 and -2, and VEGF-B, all of which have been associated with abnormal vascular remodeling [2].

### 3.2. Mathematical Model

**Figure 1:**
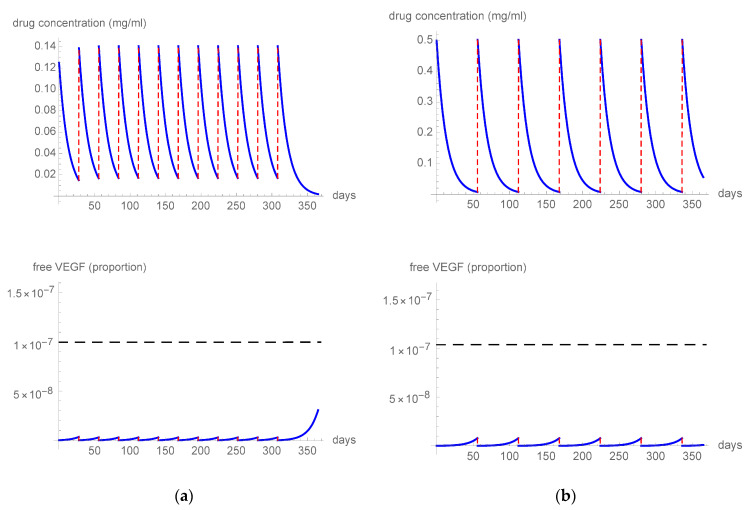
The figure describes the intravitreal drug concentration and unbound VEGF proportion in a patient treated with (**a**) q4 0.5 mg or (**b**) q8 2 mg aflibercept. The horizontal dashed line indicates the free VEGF threshold level.

**Figure 2:**
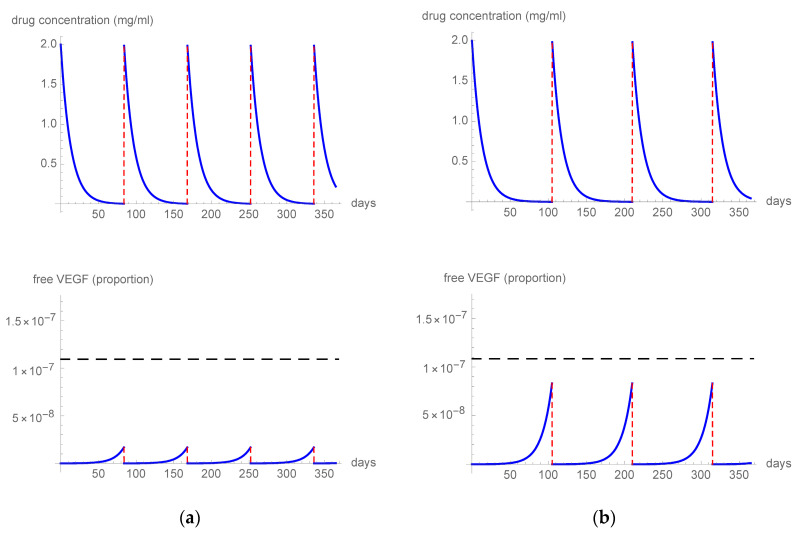

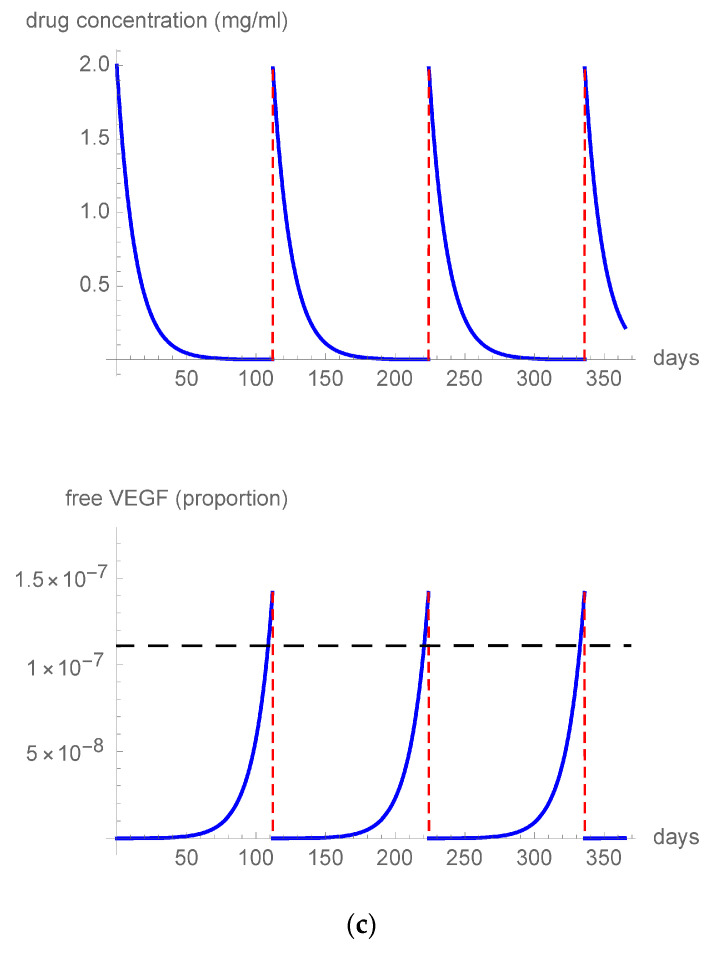
The figure describes the intravitreal drug concentration and unbound VEGF ratio in a patient treated with q12 (**a**), q15 (**b**), or q16 (**c**) 8 mg aflibercept. The horizontal dashed line indicates the free VEGF threshold level.

The first simulation (Figure 1a) tested a q4 (one treatment every 4 weeks) 0.5 mg aflibercept regimen and it showed that the free VEGF levels remained below the threshold, resulting in a constant VEGF blockade during inter-treatment intervals. In a second simulation (Figure 1b), we imputed the parameters of a q8 (one treatment every 8 weeks) 2 mg regimen, which also resulted in the free VEGF levels remaining consistently below the threshold, providing strong inhibition of VEGF for the whole period. The third simulation (Figure 2) tested the effect of 8 mg aflibercept in three different regimens: q12 (one treatment every 12 weeks) (Figure 2a), q15 (one treatment every 15 weeks) (Figure 2b), and q16 (one treatment every 16 weeks) (Figure 2c). The 8 mg q12 and q15 regimens resulted in the free VEGF levels constantly remaining under the threshold level of 0.001%. In contrast, some free VEGF spikes above the threshold level were observed, starting at day 105 after treatment, when analyzing the q16 regimen.

## 4. Discussion

Age-related macular degeneration represents the leading cause of visual impairment and irreversible blindness among individuals in developed countries [1]. Neovascular AMD is characterized by the growth of abnormal, fragile blood vessels that arise from the choriocapillaris. As the disease progresses, these new blood vessels leak fluid, cause fibrosis and the distortion of the retinal architecture, leading to permanent and irreversible damage to the macula. Left untreated, neovascular AMD can have a devastating impact on an individual’s quality of life [2,19]. Anti-VEGF agents have been proven to be effective and safe in treating neovascular AMD, significantly impacting patient prognosis and reducing the global prevalence of blindness [3,4,20,21,22]. Nonetheless, there are several drawbacks to their use, with the most salient being the necessity for recurrent intravitreal administration [23]. This requirement imposes an increased burden on patients, caregivers, and healthcare systems. In light of this, there is a need to develop alternative approaches to improve treatment durability in order to maintain sustained efficacy with fewer injections.

One strategy is developing drugs that allow higher molar intravitreal dosing, such as brolucizumab, a single-chain variable fragment (scFv) composed of fused variable regions from both the heavy- and light-chain domains of immunoglobulins. As the smallest functional component of an antibody, the Fv region, brolucizumab’s molecular weight is 26 kDa due to the absence of the Fc region, thus facilitating elevated molar dosing. Moreover, brolucizumab can achieve a concentration of up to 120 mg/mL, allowing for a single 50 μL intravitreal injection to deliver a clinical dosage of 6 mg of the drug [24,25]. Investigated in the HAWK and HARRIER trials, brolucizumab demonstrated similar efficacy to aflibercept, but allowed for 12-week intervals in most patients [26,27].

Other studies reported that brolucizumab was also beneficial for eyes that were unresponsive to available anti-VEGF drugs [28]. Nonetheless, the observed increased prevalence of intraocular inflammatory events within the HAWK and HARRIER studies, specifically pertaining to retinal vascular inflammation and occlusion, has elicited certain apprehensions regarding safety. Despite their lower incidence in real-world settings, vision loss events still occurred [29,30,31].

Another approach is using multi-targeted compounds, such as faricimab, a bispecific antibody inhibiting both VEGF-A and Ang-2 [32]. Ang-2 is implicated in inflammation and blood vessel stabilization/destabilization, making dual-targeting agents more comprehensive in treating neovascular AMD [33,34,35,36]. The phase 3 TENAYA and LUCERNE trials compared faricimab with aflibercept, showing non-inferiority in terms of clinical outcomes and favorable safety profiles [33]. At week 48, approximately 80% of patients treated with faricimab were receiving injections every 12 or 16 weeks [33].

A third viable and readily accessible way to reduce the frequency of treatments is to increase the dosage of an existing anti-VEGF drug. Recently, a novel aflibercept formulation has been developed that allows for 8 mg of the drug to be concentrated in a volume of 0.07 mL, making it suitable for intravitreal administration [37,38]. Evidence from earlier studies indicates that a consistent treatment protocol is necessary for maintaining intraocular unbound VEGF concentrations below a specific threshold in order to preserve the initial visual improvements achieved during the loading phase [39]. In previous work, we proposed that the free VEGF threshold level should be 0.001% [11]. The accuracy of this inference and our model’s outcomes were confirmed by findings from Muether et al., who studied the temporal correlations between VEGF suppression, recurrence of neovascular AMD, and loss of vision [15].

The purpose of this study was to systematically investigate the changes in intravitreal drug concentrations and the corresponding proportions of free VEGF as a function of escalating aflibercept dosages. Moreover, this research aimed to assess its effectiveness in achieving a sub-threshold free VEGF proportion following multiple intravitreal injections, considering the time-dependent dynamics of this process. In pursuit of these objectives, we employed a computational simulation to scrutinize the drug dynamics and their clinical implications. Through this approach, we successfully devised a mathematical model that provides analytical solutions for the temporal variations in the free VEGF levels, taking into account the drug’s dynamics. The concentration of aflibercept within the model was informed by the incorporation of treatment schedules, thereby generating a set of functions that characterized the drug’s behavior. The cyclical nature of the treatment regimen necessitates a series-based solution, which, in turn, enables the customization of the model to accommodate distinct therapeutic approaches. By integrating these components, our model facilitates a comprehensive understanding of the drug’s impact on the free VEGF levels and offers valuable insights for optimizing individualized treatment plans in clinical practice.

Initially, we employed the developed model to simulate the repeated administration of 2 mg aflibercept, the currently marketed dosage. When the drug was administered at 8-week intervals, our findings indicated that the free VEGF proportions consistently remained below threshold levels. This finding aligns with the clinical validation observed in the VIEW trials, where bi-monthly dosing of aflibercept proved as effective as monthly ranibizumab in preserving the initial BCVA gains throughout the follow-up period [18]. Our simulation further demonstrated that extending the dosing interval to 12 weeks resulted in a free VEGF proportion that was still persistently below the threshold. Although no published studies have yet evaluated aflibercept using a fixed 12-week regimen, insights can be gleaned from the ALTAIR and ARIES trials. In both studies, patients received a loading dose comprising three monthly aflibercept injections, followed by a treat-and-extend (TAE) regimen [40,41]. This proactive treatment strategy allows for the gradual extension or reduction of injection intervals based on the achievement of anatomic and functional stability or the presence of deterioration, respectively. By titrating injection intervals in accordance with patients’ visual and morphological outcomes and making adjustments as necessary, the ALTAIR and ARIES trials showed that most patients experienced a mean last injection interval of at least 12 weeks and significant visual improvement at 96 weeks [40,41]. These clinical findings corroborate the results observed in our simulation, further strengthening the validity of our model.

Building upon our initial findings, we proceeded to simulate and analyze the modifications in drug dynamics arising from an increment in the aflibercept dosage to 8 mg. Our simulation results imply that maintaining a free VEGF proportion below the threshold level is feasible when employing an 8 mg aflibercept dosing regimen with fixed 12 or 15-week intervals. A closer examination of the data revealed that a sub-threshold free-VEGF proportion could be sustained for up to 105 days following the intravitreal administration of 8 mg of aflibercept. This observation highlights the potential benefits of a higher aflibercept dosage in achieving prolonged VEGF inhibition, which may have significant implications for optimizing treatment protocols in clinical practice.

These findings are clinically substantiated by the outcomes of the PULSAR trial, a randomized, double-masked, phase 3, active-controlled clinical investigation devised to evaluate the efficacy and safety of high-dose aflibercept (8 mg) in neovascular AMD patients [10,42,43,44]. The study’s objective was to determine if high-dose aflibercept, administered at extended intervals (12 and 16 weeks), demonstrated non-inferiority compared with the standard 2 mg dose provided every 8 weeks. The primary endpoint was the variation in BCVA after one year, with a non-inferiority margin of four letters The results of the trial showed that the primary endpoint was achieved for both the 12-week (8q12) and 16-week (8q16) arms. In the case of disease progression, the protocol included a regimen modification, with the minimum dosing interval being 8 weeks. High-dose aflibercept treatment sustained at least q12 intervals for 83% of the patients. Seventy-nine percent and seventy-seven percent of participants maintained q12 (one injection every 84 days) and q16 (one injection every 120 days) dosing intervals without the need for regimen modification, respectively. These results are in agreement with our virtual model simulations and demonstrate the efficacy and safety of high-dose aflibercept administered at longer intervals in the management of neovascular AMD.

The development of mathematical models is often necessary to study complex biological phenomena, such as the interactions between VEGF and anti-VEGF molecules in neovascular AMD. However, these models are not exempt from limitations and assumptions that can affect the reliability and applicability of their results. One of the major limitations of the present work is related to the affinity value used for the mathematical model. In our study, we relied on data from indirect assay methods, which may not accurately reflect the true interactions between VEGF and anti-VEGF molecules in vivo [16]. Additionally, the determination of KDa values in vivo is strongly platform-dependent and can vary among different drugs and individuals, further complicating the accuracy of our results. Another limitation of our model is related to the assumptions we made regarding the penetration of anti-VEGF drugs through the retinal layers and the retinal pigment epithelium. We assumed that all dosages penetrated in the same manner and that their intravitreal concentrations correlated with the drug concentration at the neovascular AMD level in the same way. However, in a real patient, the penetration of different compounds can vary depending on their molecular characteristics and the local tissue environment. Moreover, our model only focused on the proportion of free VEGF and did not consider the actual, true concentration of free VEGF in an individual patient [45]. Furthermore, our model only simulated the intravitreal concentrations of free VEGF-A165, even though other pro-angiogenic and pro-inflammatory cytokines play significant roles in neovascular AMD activity. This limitation is particularly important, given the growing evidence supporting the important role played by these cytokines and inflammatory mediators in the pathogenesis of neovascular AMD [46,47]. This limitation could affect the generalizability of our results to the real-life management of patients with neovascular AMD. Another limitation is related to the resistance that can develop to anti-VEGF drugs during therapy, which was not considered in our model. This resistance can occur due to factors such as tachyphylaxis or tolerance and can significantly affect the efficacy of aflibercept. Finally, our model did not take into account other factors that may limit its applicability, such as the non-uniform distribution of aflibercept within the vitreous and the individual variability in the real in vivo half-life of aflibercept, which can lead to a wide range of VEGF suppression times in neovascular AMD patients [48,49,50]. Overall, while our mathematical model provides valuable insights into the interactions between VEGF and different dosages of aflibercept in neovascular AMD, it is important to consider its limitations and assumptions when interpreting and applying its results to real-life clinical practice. Further research is needed to develop more accurate and reliable models that can capture the complexity of these interactions in patients with neovascular AMD.

In conclusion, despite the listed limitations, our work suggests that increasing the dose of aflibercept to 8 mg is a viable strategy for reducing the need for frequent injections when treating patients with neovascular AMD, thus substantiating the use of a fixed regimen up to every 15 weeks and above in specific eyes. Future clinical studies are warranted to confirm these findings and evaluate the efficacy of this dosing regimen in real life clinical practice.

## Data Availability

Not applicable.
